# Cold Atmospheric Plasma Improves Shear Bond Strength of Veneering Composite to Zirconia

**DOI:** 10.3390/dj9060059

**Published:** 2021-05-21

**Authors:** Oskar Bunz, Paul Kalz, Carla I. Benz, Ella A. Naumova, Wolfgang H. Arnold, Andree Piwowarczyk

**Affiliations:** 1 Department of Prosthodontics, School of Dentistry, Faculty of Health, Witten/Herdecke University, 58455 Witten, Germany; Carla.Benz@uni-wh.de (C.I.B.); Andree.Piwowarczyk@uni-wh.de (A.P.); 2 Private Practice, 45128 Essen, Germany; Paul.Kalz@uni-wh.de; 3 Department of Biological and Material Sciences in Dentistry, School of Dentistry, Faculty of Health, Witten/Herdecke University, 58455 Witten, Germany; Ella.Naumova@uni-wh.de (E.A.N.); Wolfgang.Arnold@uni-wh.de (W.H.A.)

**Keywords:** zirconium, yttrium-stabilized-zirconium, veneering composite, shear bond strength, surface treatments, air abrasion, cold atmospheric plasma, silica-coating, energy-dispersive X-ray spectroscopy

## Abstract

Chipping of veneering is the most common clinical complication for zirconia restorations. Veneering composite could be a promising alternative to renew restorations. Zirconia discs (3-YSZ) were prepared with varying surface treatments and bonded to indirect composite as follows: air abrasion and Scotchbond Universal (A/SU); air abrasion and Clearfil Ceramic Primer (A/C); air abrasion and MKZ Primer (A/M); air abrasion and Monobond Plus (A/MP); silica-coating and Scotchbond Universal (S/SU); air abrasion (AP/SU), additional cold atmospheric plasma treatment, and Scotchbond Universal. An indirect composite material was then applied to the zirconia specimens. Specimens were divided into subgroups for short-term (14 days storage at 37 °C and 5000 thermal cycles) and long-term (250 days storage and 37,500 thermal cycles) artificial aging. Shear bond strength measurement (SBS) was performed, and data were analyzed by Kruskal–Wallis-test and multiple comparison testing with Dunn’s correction (*p* ≤ 0.05). The median SBS values (MPa) of short- and long-term artificial aging were: 3.09/1.36 (A/SU); 0.77/1.43 (S/SU); 2.82/2.15 (AP/SU); 1.97/1.80 (A/C); 2.01/1.58 (A/M); and 1.70/1.68 (A/MP). For short-term artificial aging A/SU showed the highest median SBS values, whereas in the long-term trial, AP/SU showed the highest values and the difference was significant. A prolonged artificial aging decreased SBS in all groups, except S/SU. In summary, treatment with CAP can improve SBS in the long-term.

## 1. Introduction

Currently, the use of zirconium dioxide in restorative dentistry is more and more common due to its mechanical and biological behaviors, superior esthetics compared to metal-based restorations, and lower production costs compared to precious metal alloys [1,2]. It is used for the production of a wide field of applications such as veneers, crowns, fixed partial dentures, as well as cores, posts, and even implants and implant abutments [3,4]. Ceramic layering materials are able to mask the opaque appearance of zirconia [5] and most importantly, the appearance of the restoration can be individualized and customized. However, or precisely because of this, cohesive fracture of the veneering porcelain is the most common clinical complication for zirconia restorations [6,7,8,9]. The average annual chipping rates range between 1–8% [10]. It can be assumed that more restorations made of zirconium materials will be fabricated in the future. Therefore, the increased occurrence of cohesive fracture of the veneering porcelain is also to be expected.

Depending on the size of the fracture defect, the cost, time investment, and treatment plan can vary between a composite repair or manufacturing of a new restoration [11]. The latter results in an additional trauma to the tooth structure and a more costly and time consuming treatment process [12]. In the past 10 years, it has become common to use an adhesive system with a corresponding composite resin to repair the affected sites in a direct procedure. This procedure reduces time and cost issues, and also ensures adequate esthetics [13,14]. Over time it became clear that the strength of the adhesive bond and its durability between the zirconia surface and composite resin are the key factors for treatment success [15].

Various systems and experimental methods to achieve such bonds have been proposed including cold atmospheric plasma [16], selective infiltration-etching [17], and gas fluorination [18]. Still, there is no general accordance for the most applicable surface treatment method [19], although several studies suggest that surface conditioning of zirconia substrates with air-particle abrasion and 10-Methacryloyloxydecyl dihydrogen phosphate (MDP monomer) containing coupling agents results in adequate chemical bonding [20,21,22,23].

The use of cold atmospheric plasma (CAP) might also be a promising method. This treatment leads to a better wettability of the ceramic surfaces [24,25], which may lead to a deeper penetration of the adhesive and thus may result in higher bond strengths. It was already known that the combination of surface treatments with air abrasion, cold atmospheric plasma, and adhesives can cause a significant increase in shear bond strength (SBS) to different materials [26,27].

However, there is still only limited information available on how different surface conditioning methods of zirconium dioxide influence the bond strength to composite resin in short- and long-term artificial aging. Therefore, the purposes of this in vitro study were to evaluate which surface conditioning option results in the highest SBS, whether SBS decreases due to increased artificial aging, and if surface infiltration of the bonding agent and SBS are correlated.

## 2. Materials and Methods

### 2.1. Computer-Aided Design/Computer-Aided Manufacturing (Cad/Cam) Fabrication of Zirconia Specimens

For the study, 3 mol-% yttrium stabilized zirconia (3-YSZ) specimens (Lava Plus Multi XL, 3M Oral Care, Seefeld, Germany) were CAD/CAM fabricated. The milled blanks were 12.6 mm in diameter and 6.3 mm in height and then sintered according to the manufacturer’s instructions. The target size achieved was 10 mm diameter and 5 mm height.

### 2.2. Study Groups

The specimens were divided into six groups concerning the specific surface treatment and adhesive system used on them. The composition of the study groups and the materials used are illustrated in Table 1.

### 2.3. Surface Treatment

The specimens of all groups but S/SU were conditioned with 50 μm alumina (Korox 50, BEGO, Bremen, Germany) air abrasion (Table 1). A distance of 10 mm was maintained, with a 20 s exposure time and 2 bar pressure.

The S/SU group was treated differently (Table 1). Here, the surface was conditioned using Rocatec Pre and Rocatec Plus (110 μm, 2.8 bar) with the same exposure time and distance.

### 2.4. Bonding Agents

All bonding agents were selected for their suitability on ceramic surfaces and processed according to the manufacturer’s instructions. The following were used: Scotchbond Universal (3M Oral Care, Seefeld, Germany); Clearfil Ceramic Primer (Kuraray Europe GmbH, Hattersheim am Main, Germany); MKZ Primer (Bredent GmbH & Co.KG, Senden, Germany); and Monobond Plus (Ivoclar Vivadent, Schaan, Lichtenstein).

### 2.5. Treatment with Cold Atmospheric Plasma

The AP/SU group was first treated with cold atmospheric plasma (CAP) prior to the application of the bonding agent. The CAP generator kINPen med (neoplas GmbH, Greifswald, Germany) was operated with argon gas (purity 4.8), with the pressure set to 2.5 bar and the flow rate set at 5 L/min. Before starting the surface treatment, the system ran for 5 min to stabilize the plasma jet. The handpiece of the kINPen med was installed vertically in a laboratory holder to maintain a distance between plasma jet and specimen of 10 mm. The specimens were then moved steadily under the plasma jet and treated for 60 s.

### 2.6. Specimen Preparation

All groups were embedded into a custom made alignment apparatus in which the veneering composite (Sinfony, 3M Oral Care, Seefeld, Gemany) could be applied centrally through an acrylic tube (Ø = 5 mm) onto the zirconium discs. The layer was initially polymerized for 5 s. Final polymerization used 1 min of light exposure plus 14 min of light and vacuum (Visio Beta Vario, 3M Oral Care, Seefeld, Germany).

### 2.7. Artificial Aging

Specimens were stored in a remineralization solution (Aqua bidest1 mol Potassium chloride, 150 mmol Calcium chloride, 90 mmol Potassium dihydrogen phosphate) as published recently [28]. The specimens were divided into two subgroups of short- and long-term artificial aging. For short-term artificial aging, specimens were stored at 37 °C for 14 consecutive days and subjected to 5,000 thermal cycles between 5 °C and 55 °C (Thermocycler THE1000, SD Mechatronik, Feldkirchen-Westerham, Deutschland). For long-term artificial aging, samples were stored for 250 days at 37 °C and 37,500 thermal cycles.

### 2.8. Shear Bond Strength (SBS) Measurement

Afterwards specimens were fixed to a mounting jig, a shear force was applied using a universal testing machine at a crosshead speed of 1mm/min (TA.HDplus, Stable Micro Systems Ltd., Godalming, UK). Values in MPa were assigned for statistical analysis.

### 2.9. Energy-Dispersive X-ray Spectroscopy (EDS)

Two randomly chosen specimens from each group of short-term samples were embedded into a Technovit 3040 (Kulzer, Hanau, Germany) and cut in half under water cooling with an annular saw (Leica SP 1600, Wetzlar, Germany). The probe was cut from the backside to reduce false results from overheating on the cutting surface. The specimens were placed on a bracket and observed under a scanning electron microscope (SEM). The images were made under vacuum (VP = 20 Pascal) with a voltage of 20 kV and a working distance of 8.0 mm. The magnification of 500× was adjusted with the variable pressure secondary electron generation 3 (VPSE G3) and an angle selective backscattered (AsB) detector for high-contrast images.

The elemental composition of the probes was analyzed within 50 µm of the surface depths using energy-dispersive X-ray spectroscopy (EDS) with a resolution of 512 × 400 pixels.

### 2.10. Statistical Analysis

Data were tested for normal distribution and then the non-parametric Kruskall–Wallis test was applied. The multiple comparison was analyzed with an alpha level of 0.05. The evaluation, descriptive data analysis, and graphical representation were performed using GraphPad Prism (Version 8.4.3, GraphPad Software, San Diego, CA, USA).

## 3. Results

**Figure 1:**
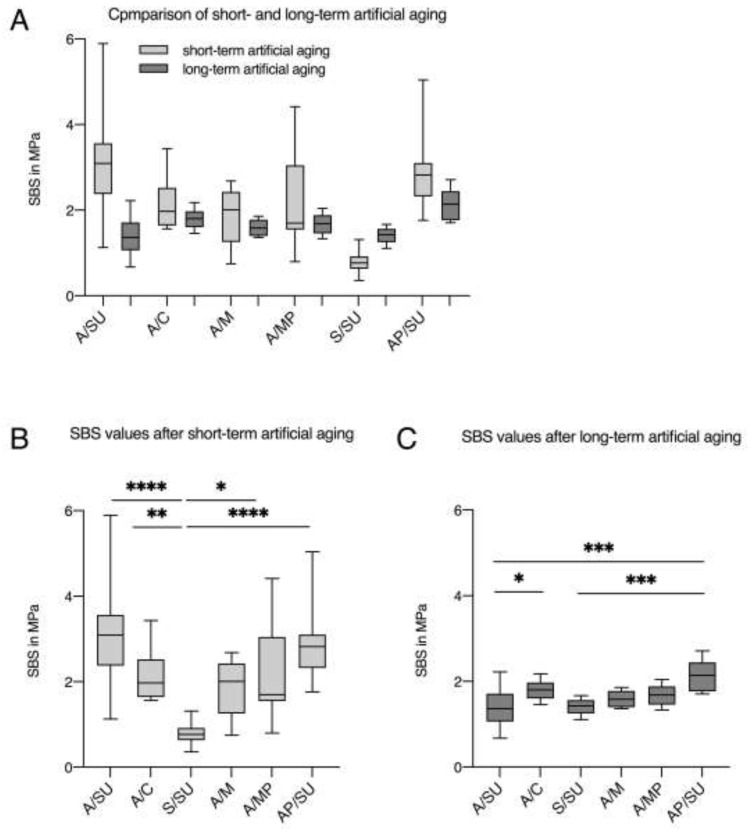
Results of SBS values after short- and long-term artificial aging. A/SU: air abrasion and Scotchbond Universal; A/C: air abrasion and Clearfil Ceramic Primer; A/M: air abrasion and MKZ Primer; A/MP: air abrasion and Monobond Plus; S/SU: silica-coating and Scotchbond Universal; AP/SU: air abrasion, additional cold atmospheric plasma treatment (CAP), and Scotchbond Universal. (**A**) Boxplots comparing SBS values in MPa after short- and long-term artificial aging. (**B**) Median values for short-term test compared by Kruskall–Wallis test * *p* < 0.05; ** *p* < 0.005; *** *p* < 0.0005; **** *p* < 0.00005. (**C**) Median values for long-term test compared by Kruskall–Wallis test * *p* < 0.05; *** *p* < 0.0005.

The investigation of SBS values after short- and long-term aging showed a distinctive pattern. All SBS values decreased after longer aging. Only the initially lower SBS values of S/SU adjusted to the other groups after prolonged aging and increased slightly (Figure 1A and Table 1).

Looking at the short-term experiments individually, it is noticeable that the median values of Scotchbond Universal after air abrasion (A/SU) and Scotchbond Universal with previous CAP treatment (AP/SU) are very similar and seem superior to the other groups. No significant difference can be found between these groups.

After prolonged artificial aging, a different picture emerged. With a median of 2.1 MPa (Table 2), AP/SU had the highest median value and achieved a significantly higher SBS value than that of A/SU (Figure 1C).

To investigate the initial penetration depth of the adhesive systems into the roughened surface of the zirconia specimens, two randomly selected test specimens were examined by means of Energy-Dispersive X-ray Spectroscopy (EDS) analysis. The carbon content was measured as an indicator for the adhesive system. This was accompanied by the decrease in zirconia percentage. The yttrium content was also determined but remained constant in all groups.

EDS analysis shows a sudden increase in carbon levels up to 36% of all groups in the surface range of 5 µm. At 5 µm below the surface up to a depth of 50 µm the carbon levels decreased slightly and reached 18–20% (data not shown).

**Figure 2:**
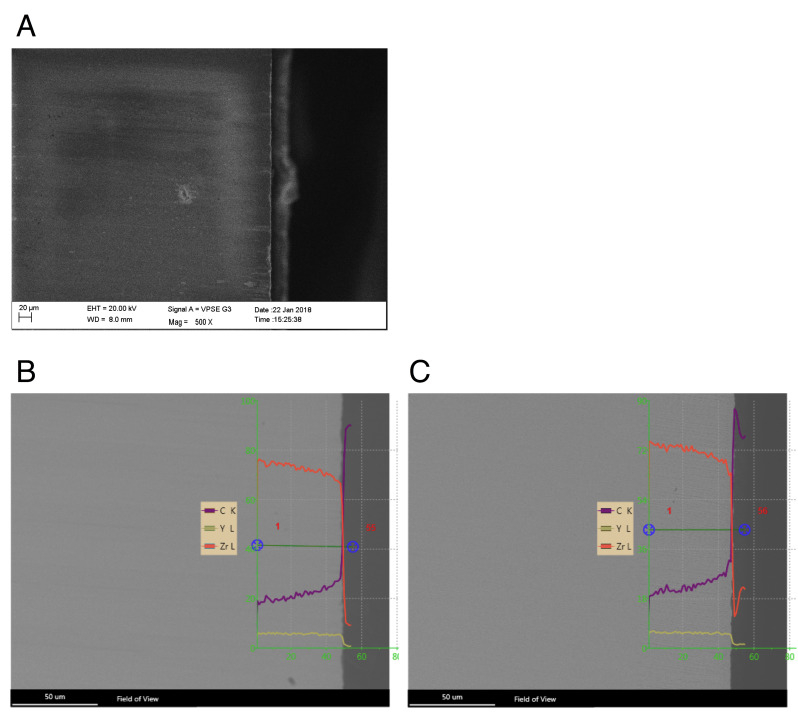
Scanning electron microscope (SEM) and energy-dispersive X-ray spectroscopy (EDS) analysis of specimen slides. (**A**) Detection image of a zirconia specimen in cross section after short-term artificial aging. EDS image of (**B**) A/SU: air abrasion and Scotchbond Universal and (**B**) AP/SU: air abrasion, additional cold atmospheric plasma treatment (CAP), and Scotchbond Universal. (**C**): Carbon, Y: Yttrium and Zr: Zirconium are shown as percentages up to a depth of 50 µm.

The specimens of the S/SU and AP/SU groups are shown here as examples (Figure 2).

S/SU showed a carbon content of 19% at the 50 µm depth, which increased to 22% at the surface. The percent zirconia decreased in the same proportion (Figure 2B).

The EDS analysis for the AP/SU group showed a carbon content of 18% at the 50 µm depth. However, this increased to a value of 27% at the surface. The percentage of zirconium decreased in a similar pattern (Figure 2C).

## 4. Discussion

In the present study, SBS values between a zirconia ceramic and an indirect composite with different surface conditioning methods in short- and long-term artificial aging were measured to assess the durability of the coatings. The SBS values presented in this study are noticeably low compared to other studies, and they are not satisfactory or sufficient for clinical use.

In some cases, values of more than 20 MPa were achieved in the adhesive bond to zirconium surfaces when MDP-containing adhesive systems were used [29,30]. Of course, in all comparisons, attention must be paid to the different parameters of the studies. SBS measurements cannot be compared with micro shear procedures nor tensile bond strength measurements, especially since the geometry of specimens are different [31]. In addition, attention must be paid to the durations and the type of artificial aging. In the present study, extremely long periods of storage and thermocycling were chosen. This may lead to significantly reduced SBS values, but at the same time longer observation periods can lead to higher clinical safety.

Mechanical retention to ceramics and metal is necessary, otherwise adhesion of acrylic polymers to these materials is insufficient. Specifically, chemical bonding to zirconia is difficult since there is no glass matrix. The advantages of MDP and silane have been described many times in other studies, at least for bonding to luting cements [32] and composites [33]. Both reviews see the need for pre-treatment via air abrasion.

Roughness such as fissures or pits form microscopic overhangs, which increase the surface area and can improve the adhesion. On the other hand, an excessively fissured surface, if insufficiently wetted, can cause air pockets that drastically reduce the bond strength. Especially at voids, thermal cycling conditions and mechanical loading can cause increased stress. This can then lead to a fracture gap between the adhesive and the cavity, causing detachment [34].

One solution would be to choose an adhesive with the lowest possible viscosity, as the lowest possible viscosity could ensure a more reliable wetting, since better wetting leads to more effective penetration into the surface irregularities due to capillary forces.

Lowing the surface tension is another possibility. This can be achieved, for example, by using cold atmospheric plasma (CAP).

CAP is generated when a gas is partially ionized in a high-voltage electric field [27]. The resulting charged particles induce effects on the treated surface, such as organic residue removal, micro-etching, surface activation, and crosslinking [35,36].

It has been reported that CAP increases the adhesive forces after conventional air abrasion with alumina particles without affecting the surface finish. The use of only CAP reduces the risk of fissuring and cracking [37].

It is believed that the reactive atoms break the weakly stable bonds on the surface and expose the polar bonds [38,39]. These can now form new chemical bonds by forming radicals from oxygen and nitrogen [38,40].

In our study, we could not show any enhancement in adhesion through CAP initially. Nevertheless, prolonged artificial aging showed a certain superiority of the additional CAP treatment.

Further studies are needed to investigate whether the reduction in surface tension can lead to a better bond strength of veneering composite to zirconia. It should also be investigated whether CAP treatment could also eliminate the need for an adhesive system.

## 5. Conclusions

Within the limitations of an in vitro study, the results presented indicate that air abrasion with alumina and application of a MDP-based adhesive system provides the highest SBS to zirconia. The addition of CAP treatment resulted in the highest long-term durability of SBS.

## Figures and Tables

**Table 1:** Composition of test groups and the materials used. Surface treatments, bonding materials, and veneering composites are displayed to the corresponding groups. A/SU: air abrasion and Scotchbond Universal; A/C: air abrasion and Clearfil Ceramic Primer; A/M: air abrasion and MKZ Primer; A/MP: abrasion and Monobond Plus; S/SU: silica-coating and Scotchbond Universal; AP/SU: air abrasion, additional cold atmospheric plasma treatment (CAP) and Scotchbond Universal.

Group	A/SU	A/C	A/M	A/MP	S/SU	AP/SU
Surface Treatment	Air Abrasion with 50 μm Alumina Oxide (Al_2_O_3_)				Silica-coating	Air Abrasion with 50 μm Alumina Oxide (Al_2_O_3_) and Cold Atmospheric Plasma
Bonding Material	Scotchbond Universal, 3M Oral Care, Germany	Clearfil Ceremic Primer, Kuraray Europe, Germany	MKZ Primer, Bredent, Germany	Monobond Plus, Ivoclar Vivadent, Liechtenstein	Scotchbond Universal, 3M Oral Care, Germany	Scotchbond Universal, 3M Oral Care, Germany
Veneering Composite	Sinfony, 3M Oral Care, Gemany					

**Table 2:** Descriptive data analysis for short- and long-term artificial aging. A/SU: air abrasion and Scotchbond Universal; A/C: air abrasion and Clearfil Ceramic Primer; A/M: air abrasion and MKZ Primer; A/MP: air abrasion and Monobond Plus; S/SU: silica-coating and Scotchbond Universal; AP/SU: air abrasion, additional cold atmospheric plasma treatment (CAP), and Scotchbond Universal. Minimum; 25% percentile; median; 75% percentile; maximum; range and number of values are listed in MPa.

Short-Term	A/SU	A/C	A/MP	A/M	S/SU	AP/SU
Minimum	1.12	1.56	0.79	0.74	0.35	1.76
25% Percentile	2.37	1.64	1.55	1.25	0.63	2.32
Median	3.09	1.97	1.69	2.00	0.76	2.82
75% Percentile	3.56	2.52	3.05	2.42	0.91	3.10
Maximum	5.89	3.43	4.41	2.68	1.31	5.04
Range	4.76	1.86	3.62	1.93	0.95	3.28
Number of values	12	12	12	12	12	12
Long-Term	A/SU	A/C	A/MP	A/M	S/SU	AP/SU
Minimum	0.67	1.45	1.33	1.36	1.10	1.70
25% Percentile	1.06	1.60	1.45	1.39	1.25	1.76
Median	1.36	1.80	1.68	1.58	1.42	2.14
75% Percentile	1.71	1.97	1.88	1.77	1.56	2.44
Maximum	2.22	2.17	2.04	1.85	1.66	2.71
Range	1.55	0.72	0.71	0.49	0.55	1.00
Number of values	10	10	10	10	10	10

## Data Availability

The data presented in this study are available on request from corresponding author.
